# Sleep Health Analysis Through Sleep Symptoms in 35,808 Individuals Across Age and Sex Differences: Comparative Symptom Network Study

**DOI:** 10.2196/51585

**Published:** 2024-06-11

**Authors:** Christophe Gauld, Sarah Hartley, Jean-Arthur Micoulaud-Franchi, Sylvie Royant-Parola

**Affiliations:** 1 Hospices Civils de Lyon Lyon France; 2 Sleep Center APHP Hôpital Raymond Poincaré Université de Versailles Saint-Quentin en Yvelines Garches France; 3 Réseau Morphée Garches France; 4 Services of Functional Exploration of the Nervous System University Sleep Clinic University Hospital of Bordeaux Bordeaux France; 5 Unité Sommeil, Addiction, Neuropsychiatrie Centre national de la recherche scientifique Unité Mixte de Recherche–6033 Bordeaux France

**Keywords:** symptom, epidemiology, age, sex, diagnosis, network approach, sleep, sleep health

## Abstract

**Background:**

Sleep health is a multidimensional construct that includes objective and subjective parameters and is influenced by individual sleep-related behaviors and sleep disorders. Symptom network analysis allows modeling of the interactions between variables, enabling both the visualization of relationships between different factors and the identification of the strength of those relationships. Given the known influence of sex and age on sleep health, network analysis can help explore sets of mutually interacting symptoms relative to these demographic variables.

**Objective:**

This study aimed to study the centrality of symptoms and compare age and sex differences regarding sleep health using a symptom network approach in a large French population that feels concerned about their sleep.

**Methods:**

Data were extracted from a questionnaire provided by the Réseau Morphée health network. A network analysis was conducted on 39 clinical variables related to sleep disorders and sleep health. After network estimation, statistical analyses consisted of calculating inferences of centrality, robustness (ie, testifying to a sufficient effect size), predictability, and network comparison. Sleep clinical variable centralities within the networks were analyzed by both sex and age using 4 age groups (18-30, 31-45, 46-55, and >55 years), and local symptom-by-symptom correlations determined.

**Results:**

Data of 35,808 participants were obtained. The mean age was 42.7 (SD 15.7) years, and 24,964 (69.7%) were women. Overall, there were no significant differences in the structure of the symptom networks between sexes or age groups. The most central symptoms across all groups were nonrestorative sleep and excessive daytime sleepiness. In the youngest group, additional central symptoms were chronic circadian misalignment and chronic sleep deprivation (related to sleep behaviors), particularly among women. In the oldest group, leg sensory discomfort and breath abnormality complaint were among the top 4 central symptoms. Symptoms of sleep disorders thus became more central with age than sleep behaviors. The high predictability of central nodes in one of the networks underlined its importance in influencing other nodes.

**Conclusions:**

The absence of structural difference between networks is an important finding, given the known differences in sleep between sexes and across age groups. These similarities suggest comparable interactions between clinical sleep variables across sexes and age groups and highlight the implication of common sleep and wake neural circuits and circadian rhythms in understanding sleep health. More precisely, nonrestorative sleep and excessive daytime sleepiness are central symptoms in all groups. The behavioral component is particularly central in young people and women. Sleep-related respiratory and motor symptoms are prominent in older people. These results underscore the importance of comprehensive sleep promotion and screening strategies tailored to sex and age to impact sleep health.

## Introduction

Sleep health is a multidimensional construct that includes objective and subjective parameters and is influenced by individual sleep-related behaviors [1] and sleep disorders, measurable by a set of techniques ranging from psychometry [2,3] to mobile devices [4,5]. Sleep health is not merely the absence of sleep disorder symptoms; it also includes factors such as sleep disturbance and satisfaction with sleep [6,7]. Moreover, sleep health considers the importance of sleep behaviors reinforcing both the homeostatic (duration of sleep) and the circadian (regularity and time of sleep) system that underpin sleep quality [8]. Good-quality sleep is important for overall health [9], but sleep is influenced both by age and by sex [10-12].

Sleep architecture evolves from birth to old age, with changes in the macro- and microstructure that are underpinned by neuroanatomical changes in both homeostatic and circadian systems [13]. More precisely, sleep evolves across the lifespan, with the development of a 24-hour circadian rhythm, a progressive reduction in daytime naps, and a gradual reduction in the quantity of slow-wave sleep (N3) in the early years. From the fifth decade onward, advanced sleep timing, longer sleep onset latency, shorter nighttime sleep potentially compensated by daytime naps, increased wake after sleep onset (WASO), increased fragmentation, a lower threshold for wake from sleep due to external stimuli, reduced N3, and consequently increased light sleep (N1 and N2) from the fifth decade are seen [11,12,14-22]. These later changes are accompanied by changes in the microstructure of sleep with a loss of the homeostatic drive expressed as reductions in slow-wave amplitude and density [23] and modifications in spindles and K-complexes [24,25]. Neuronal loss with age underpins changes in both homeostatic and circadian systems. Changes in spindles are potentially related not only to a loss of the hippocampal gray matter [26] but also to a loss of the functional integrity of corticothalamic loops [27]. A loss of suprachiasmatic nucleus (SCN) neurons, notably vasoactive intestinal peptide (VIP)–expressing ones, may contribute to changes in the circadian rhythm [28].

Sex-determined differences in the sleep macro- and microstructure are detectable from an early age, with differences in sleep disorders increasing from early puberty onward [29]. Certain sleep disorders are more frequent in men (eg, obstructive sleep apnea syndrome) [30], while others are more frequent in women (eg, insomnia disorder) [31], and these differences modulate over the lifespan. Concerning the impact of sex according to age, biological sex markedly affects sleep, and differences are detectable in newborns, with increased divergence at puberty [29]. Changes in sleep with age are modulated by sex: the marked reduction in slow-wave sleep in men over 70 years does not affect women, although reductions in rapid eye movement (REM) sleep are seen in both [12]. Numerous sex-related differences are seen at an anatomical level: larger male-specific declines are seen in galanin-expressing neurons, orexin neurons, neuromelanin-sensitive neurons in the locus coeruleus (LC), and VIP-expressing neurons in the SCN. Women report subjective changes in sleep related to menopause, with increased insomnia and reduced sleep quality [32], although this is difficult to demonstrate objectively via polysomnography [33]. Biological sex also affects sleep disorders: obstructive sleep apnea is more common in men [30] and insomnia in women [31]. In addition, sleep behaviors may vary between men and women: as circadian timing is phase-advanced in women [34], women have earlier bedtimes [35] and an earlier chronotype, although this reverses after the age of 40 years [36].

The multifactorial components contributing to sleep health are adapted to network analysis for several reasons. First, unlike a certain number of other models limiting the number of variables analyzed (eg, due to an increase in the type 1 error), symptom network analysis offers an original way to model the interactions between numerous sleep-related clinical variables according to other sociodemographic ones. In this way, it offers a more holistic view of sleep health. Second, traditional studies often examine symptoms individually, looking at their interconnected impacts. This computational network analysis not only allows visualization of the relationships between these variables but also helps identify the strength of those mutual relationships. The analysis of these interactions seems particularly important in the context of sleep health, whose mutual influencing factors have been largely undetermined until now. Third, identifying the most influential symptoms across varying age and sex demographics, by considering a large number of variables and the interactions between them, provides a clearer roadmap for health care and public health interventions. Finally, symptom network analysis has the potential for sleep health promotion at both a local and a global scale by identifying screening strategies that are sensitive to the nuanced differences across populations.

In our previous work using a data-driven network analysis approach in a large data set including sleep disorders, sleep disturbances, sleep-related behaviors, and comorbidities as variables, the most central variables were a complaint of nonrestorative sleep, excessive daytime sleepiness, circadian irregularity, and chronic sleep deprivation [37]. The latter 2 variables are linked to the circadian system and the homeostatic system, respectively, whereas the complaint of nonrestorative sleep, or not feeling refreshed on wakening, is a relatively little studied concept compared to its potential importance for patients [6,38,39]. Finally, the complaint of daytime sleepiness can be considered a consequence of poor sleep, and as such, its centrality is unsurprising, given its importance for sleep physiology and for its historical and methodological centrality in the construction of sleep classifications and its relevance in clinical practice [40-43].

Given the known influence of sex and age on sleep health, extending our analysis to consider these variables is highly pertinent as network analysis explores sets of mutually interacting symptoms. It can thus detect whether the differences between groups (eg, of sex or age) may or may not be explained by some specific symptoms. Thus, although group comparison based on frequency statistics only provides an index of difference (eg, a *P* value), network analysis provides insight into which factors drive differences.

The objective of this work was to study the centrality of symptoms and to compare age and sex differences in the experience of sleep using a cross-sectional approach of comparative network analysis in a population that feels concerned by their sleep. First, we aimed to analyze the relationships between the components of these sleep networks between 2 populations, men, and women. Second, we studied these relationships according to different age classes. For these 2 analyses (sex and age), we studied the potential differences in centrality (ie, the importance), predictability, and connections between different factors related to sleep symptoms, sleep behavior, and the effects of sleep.

## Methods

### Data Collection

The Réseau Morphée is a nonprofit organization funded by the French government (regional health authority), aiming to improve patient care pathways in sleep health. In 2017, the new patient care pathway was launched, offering a comprehensive online questionnaire as the initial port of entry. The questionnaire looks at 5 domains: sociodemographic data (age and sex), symptoms of sleep disorders, sleep disturbances, sleep behaviors, and comorbidities [40]. In total, 39 variables were analyzed. We prioritized data extraction based on clinical relevance, guaranteeing it aligned with both clinical and public health relevance (and reinforcing by analysis of robustness). We also provided an example of the analyses by adding a variable (the BMI) in order to discuss such an addition and its implications. Data from 3 validated scales were included: the Epworth Sleepiness Scale (ESS; excessive daytime sleepiness score<10) [44], the Insomnia Severity Index (ISI; clinical insomnia score>14) [45], and the Hospital Anxiety and Depression Scale for anxiety (HADS-A) and depression (HADS-D) (scores>10 for each) [46].

### Ethical Considerations

Patients were informed about the use of their data for epidemiological research when data were originally collected. Informed consent was obtained from all participants included in the study. The study was approved by the scientific committee of the Réseau Morphée and by 2 patient associations, the Sommeil et Santé and the France Insomnie. As a noninterventional study (MR004), this research was approved by the Commission Nationale Informatique et Liberté (CNIL; approval number: 8013081; date of approval: December 19, 2016). Only data from participants over the age of 18 years were included in the analysis. The anonymization of data provides confidentiality and total respect for the privacy of participants. No publicity was used to attract participation. No financial compensation was provided for participating in the study.

### Participants

Participants were French-speaking members of the general population who filled in an online questionnaire aimed at people concerned about their sleep. This population does not present people who already experience sleep problems but rather people who are sufficiently concerned about their sleep to use the Réseau Morphée online questionnaire. They were not in contact with sleep services and thus did not have a formal diagnosis of their sleep problems. We included all respondents over 18 years old from March 1, 2017, to September 9, 2020. All participants in our study speak French, but all may not be currently residing in France. Regarding demographic data, we collected the sex and age of each participant. As specified later in the *Limitations* section, we considered the person’s sex as assigned at birth, not gender as defined by the person, and we chose to use the terms “men” and “women.” The entire sample was divided into 4 age groups: 18-30, 31-45 years, 46-55, and >55 years. Age group stratification was determined by known sociodemographic boundaries in France: the 18-30–year group is either still in education or in the early stages of their careers and less likely to have children (the mean childbearing age in France was 30.9 years in 2021 according to the [47]). Participants aged 31-45 years are settled in their careers and may have children. The 46-55–year group was chosen specifically to isolate the period of the menopause, during which sleep complaints in women markedly increase [32]. We provided the number of men and women (sex), the mean (SD) of age, and the BMI for the ESS, the ISI, the HADS-A, and the HADS-D, and we detailed these measurements by age group.

### Network Estimation, Inferences, Robustness, and Comparison

The main aim of this network analysis was to analyze a set of 39 variables described by a large population divided into several subpopulations by age and sex to compare the subpopulations. To this end, 6 networks were constructed for comparison: a men’s network, a women’s network, and a network for each age group (18-30, 31-45, 46-55, and >55 years).

In this methodology, the correlations between items were understood as a network in which each item represents a node and where correlations between symptoms are connections (or edges) between nodes. A connection between any 2 symptoms was said to exist if those symptoms were present in the same individual. Symptoms of 2 different disorders were connected if the symptoms were present in different individuals. We followed the network guidelines for computational analysis of network properties [48].

#### Network Estimation

Network estimation was conducted with the Ising Model, which is, to date, the state-of-the-art network model used in psychopathology research for binary data (see Multimedia Appendix 1 [49-69] for details on symptom networks) associated with a regularization technique called graphical least absolute shrinkage and selection operator (LASSO) [59,60]. The final model was chosen by using the extended Bayesian information criterion (EBIC) [61].

The network was graphically represented according to the Fruchterman-Reingold algorithm, where variables with stronger or more connections are placed closer to each other [62]. Nodal predictability was calculated based on models derived from mixed graphical models (MGMs) [63,70] and graphically represented as a pie chart in the ring around each variable. Predictability has a value between 0 and 1, provided on the basis of the variance of the prediction error calculated according to the *R*^2^ of the MGM. It refers to how well a given node in the network can be predicted by all remaining nodes. It thus shows how relevant edges are (eg, a node may be connected to many other nodes, but if these only explain only 1% of its variance, it is unlikely that this node is relevant). This has further implications: for example, designing an intervention to affect certain nodes or detecting where data are lacking (eg, when parts of the network are little influenced by related nodes and thus must depend on external factors). In clinical practice, the predictability of a symptom indicates “whether an intervention on that symptom through the symptom network is promising” [70]. The predictability of sexes and age groups is given in Multimedia Appendix 2.

In agreement with previous work [37] and the main international sleep-wake classifications [71,72], as well as to facilitate the reading of the graphic representations, we classified elements from the questionnaire into 8 groups: hypersomnia symptoms, insomnia symptoms, respiratory symptoms, motor symptoms, psychiatric symptoms, sleep disturbance, sleep behaviors, and comorbidities. These groupings had no impact on the main results of the study and were only given for data visualization purposes.

#### Network Inferences

The most clinically relevant network inferences correspond to centrality measures [64]. Centrality measures are important for identifying symptoms that play a crucial role in connecting all the symptoms of a network [65]. Nodes with high centrality index measures represent variables that are highly connected to other variables. Precisely, 4 measures are classically described: strength, closeness, betweenness, and expected influence [66]. The strength of a node computes the degree to which it is connected with all the other nodes of the network [67]. The 3 other centrality measures are described in Multimedia Appendix 1. The centrality strengths of age groups and sexes were plotted and are detailed in Multimedia Appendix 2. The color (red=0, green=1) and bar plot of each symptom provide visual insight into the *P* value. Symptoms were sorted in descending order of strength and predictability.

#### Network Comparisons

The networks were systematically compared between men and women and between the 4 age groups (18-30, 31-45, 46-55, and >55 years). Cross-age and cross-sex networks were compared between each other with a network comparison tool, providing significance indices for the overall structures of the networks and allowing us to evaluate the differences between each network.

We used the network comparison test (NCT) [73], a 2-tailed permutation test that examines differences between 2 networks. This test analyzes the difference in terms of relationships between (1) variables connections (“Are the connections between variables different between the 2 networks?”) and (2) centrality measures, named global strength (“Is the centrality of the variables different between the 2 networks?”). The NCT therefore presented 2 results: a comparison of connections between variables and a comparison in terms of centrality. *P*<.05 was considered a statistically significant difference.

There is a potential risk of loss of power when the samples are not of equal size [61]. To deal with our unequal sample, we subsampled the larger data set to the size of the smaller data set 5 times. This meant we ran the NCT 5 times, with 2000 replications for each comparison [74]. We presented the average of the results without adjusting the *P* values for multiple testing.

Moreover, to offer inferences on the comparison between networks at the level of each symptom, we computed the local symptom-by-symptom correlations between the nodes of the 2 networks, with a *P* value resulting from the permutation test concerning differences both in global strength and in edges weights. Local symptom-by-symptom correlations (*P* value based on permutations) between the strength by sex and by age are given in Multimedia Appendix 3. Local symptom-by-symptom correlations (*P* value based on permutations) between connections by sex and by age are also given in Multimedia Appendix 3. The color (red=0, green=1) and bar plot of each symptom provide visual insight into the *P* value. The symptoms were sorted according to the order of the figures in the paper. However, we only discussed the most relevant relationships rather than variables systematically significantly associated, which should only be interpreted according to all the other relationships of the network.

#### Network Robustness

To verify that the number of participants was adequate to perform such a network analysis, we studied the robustness of the network using bootstrap analysis (N=2000 iterations) [68,69]. The robustness of the results was tested on a centrality measure, the strength (ie, we checked the stability of edges and centrality measures). We used a case-dropping subset to assess the stability of this centrality measure, providing a centrality stability correlation coefficient (ie, correlation stability [CS] coefficient; we computed how well the order of centralities was retained after observing only a subset of the data): the CS coefficient (ie, the maximum proportion of participants that could be dropped, while maintaining 95% probability that the correlation between centrality metrics from the full data set and the subset data was at least 0.70). Based on a simulation study [68,69], a minimum CS coefficient of 0.25 was recommended.

All analyses and graphical visualizations were performed in *R* version 4.2.2 (*R* Foundation for Statistical Computing). Generative artificial intelligence (AI) was not used at any point.

## Results

### Participant Details

Table 1 provides details of the study sample. There were twice as many women (n=24,964, 69.7%) as men (n=10,844, 30.3%). The average age of the entire sample was 42.7 (SD 15.7) years, positioned in the studied interval of 31-45 years. The BMI of 24.7 showed an average healthy weight (≤24.9). Regarding the diagnoses associated with the entire sample, the average ESS score of 9.47 (SD 4.95) was below the threshold (≤15). However, the average ISI score of 16.34 (SD 5.24) was above the threshold (>15 for the entire sample and all subgroups). Likewise, the average HADS-A score of 9.58 (SD 3.97) and the average HADS-D score of 6.56 (SD 3.97) were above the threshold of 7 and 5, respectively, for the entire sample and all subgroups.

**Table 1:** Description of the French-speaking adult population (N=35,808) participating in this study.

Characteristics			All participants		Age groups (years)						
					18-30 (n=9051)		31-45 (n=12,129)		46-55 (n=7164)		>55 (n=7464)
**Sex, n (%)**											
	Men	10,844 (30.3)		2259 (25.0)		3661 (30.2)		2240 (31.3)		2684 (36.0)	
	Women	24,964 (69.7)		6792 (75.0)		8468 (69.8)		4924 (68.7)		4780 (64.0)	
Age (years), mean (SD, 95% CI)			42.7 (15.7, 42.75-42.92)		—^a^		—		—		—
BMI, mean (SD, 95% CI)			24.7 (5.12, 19.43-27.21)		24.44		24.59		24.88		24.87
ESS^b^ mean (SD, 95% CI)			9.47 (4.95, 9.47-9.53)		9.81		9.81		9.64		8.36
ISI^c^, mean (SD, 95% CI)			16.34 (5.24, 16.34-16.39)		15.69		16.57		16.85		16.25
HADS-A^d^, mean (SD, 95% CI)			9.58 (3.97, 9.58-9.62)		10.01		9.91		9.37		8.70
HADS-D^e^, mean (SD, 95% CI)			6.56 (3.97, 6.56-6.60)		6.37		6.82		6.67		6.25

^a^Not applicable.

^b^ESS: Epworth Sleepiness Scale.

^c^ISI: Insomnia Severity Index.

^d^HADS-A: Hospital Anxiety and Depression Scale for anxiety.

^e^HADS-D: Hospital Anxiety and Depression Scale for depression.

### Sleep Health Network Analysis

#### Network Estimation

**Figure 1:**
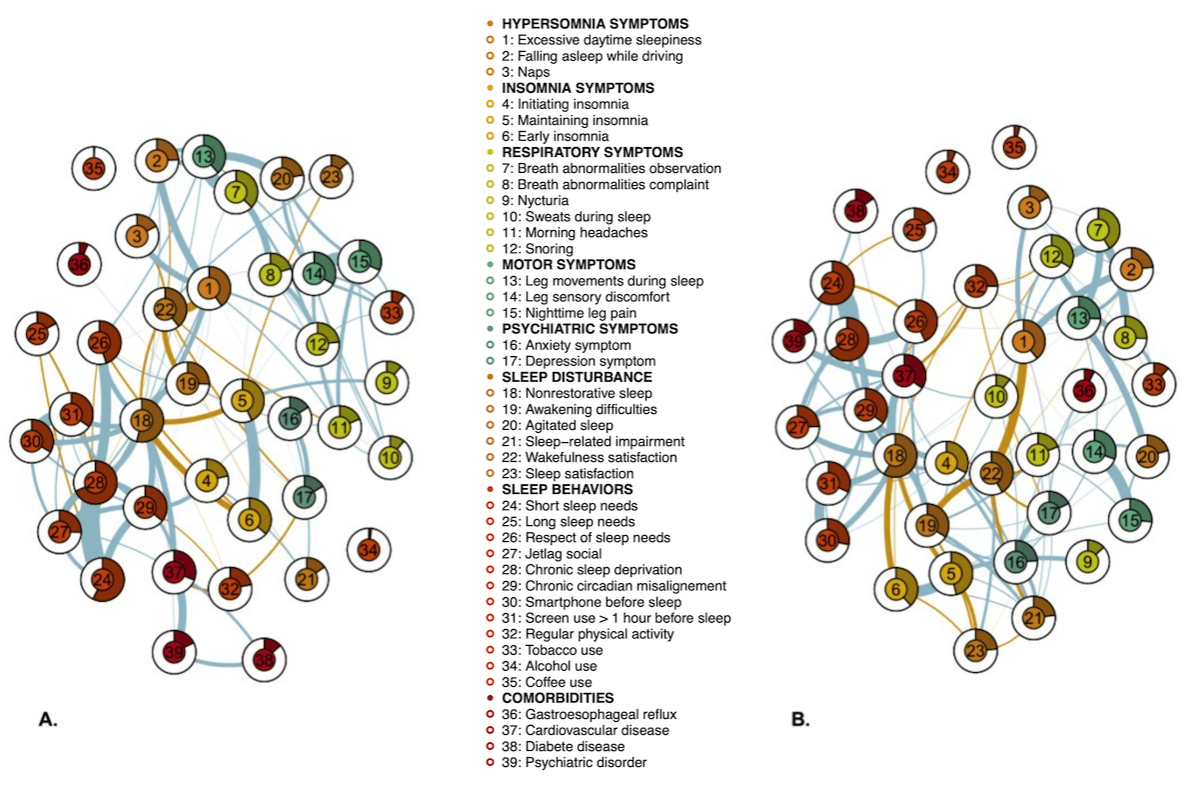
Sleep networks related to sex groups of a French-speaking adult population concerned about their sleep (N=35,808). (A) Women’s network. (B) Men’s network. Blue edges (connections) represent the positive associations between variables and orange edges the negative associations. The thickness of the line represents the level of correlation between 2 variables. The predictability of the nodes is depicted as a pie chart in the rings around the nodes: the area in the outer ring of a node represents the percentage of variance of the node that is explained by all neighboring nodes. Color groupings are only given for data visualization purposes. A higher-resolution version of this image is available in Multimedia Appendix 4.

Each network contained 39 nodes (items) and 741 edges (connections), with a mean weight of 0.011 (SD 0.005). Figure 1 presents results of the sleep health network analysis related to sex groups, with negative edges. The predictability of sexes and age groups is given in Multimedia Appendix 2.

**Figure 2:**
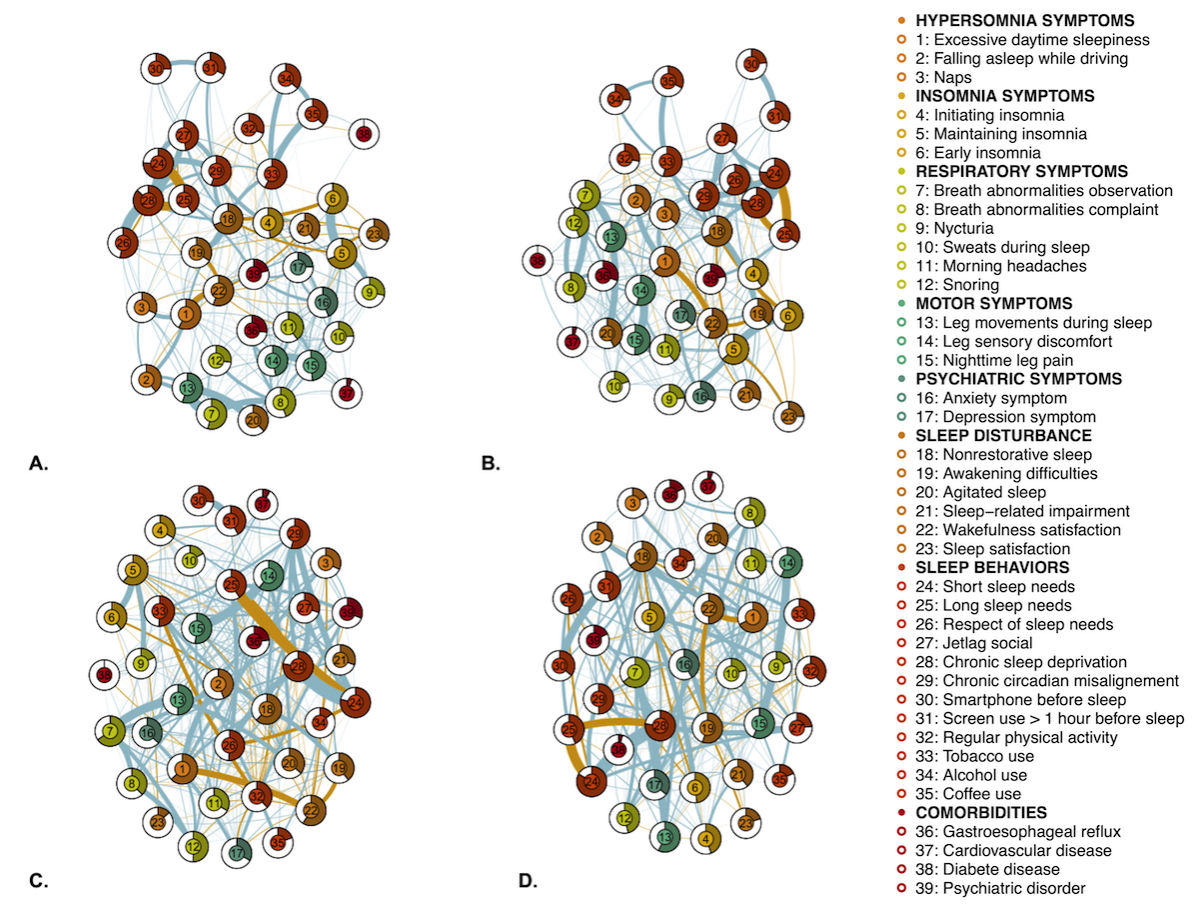
Sleep networks related to the 4 age groups of a French-speaking adult population concerned about their sleep (N=35,808). (A) 18-30 years, (B) 31-45 years, (C) 46-55 years, and (D) >55 years. Blue edges (connections) represent the positive associations between variables and orange edges the negative associations. The thickness of the edges represents the level of correlation between 2 variables. The predictability of the nodes is depicted as a pie chart in the rings around the nodes: the area in the outer ring of a node represents the percentage of variance of the node that is explained by all neighboring nodes. Color groupings are only given for data visualization purposes. A higher-resolution version of this image is available in Multimedia Appendix 4.

Results of the sleep health network analysis related to age groups are shown in Figure 2. In the figure, the 2-to-2 relationships, conditional on all other relationships between nodes in the network, can be visualized.

We first deduced from the graphical presentation which node-by-node correlations were the most relevant. In the women’s network, it was interesting to see that nonrestorative sleep was strongly negatively associated with early insomnia (*r*=–0.2). Similarly, in the men’s network, excessive daytime sleepiness was strongly negatively correlated with wakefulness satisfaction (*r*=–0.26) but positively correlated with falling asleep while driving (*r*=0.23) or with naps (*r*=0.2). Across the different age group networks, a number of relationships were consistently found, for example, between the symptoms nonrestorative sleep and awakening difficulties (*r*=0.39, 0.39, 0.36, and 0.38 in the 18-30–, 31-45–, 46-55–, and >55-year age groups, respectively, in ascending order), as well as weak relationships between breath abnormality observation and nighttime leg pain (*r*=0.12, 0.08, 0.01, and 0.1 in the 18-30–, 31-45–, 46-55–, and >55-year age groups, respectively, in ascending order).

**Figure 3:**
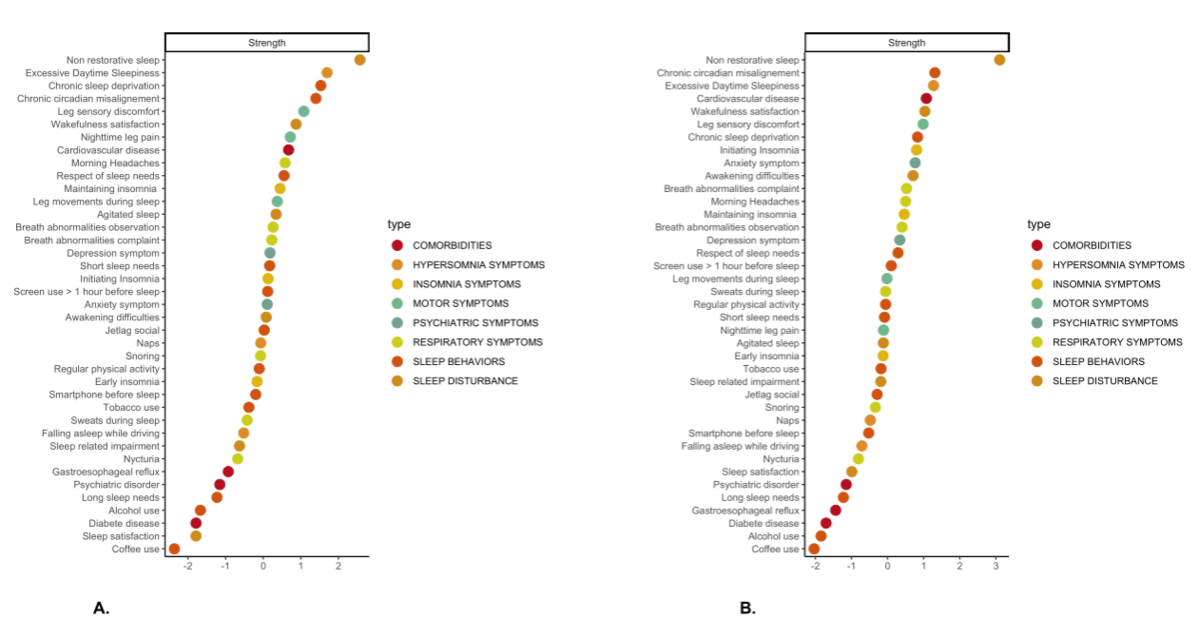
Centrality measure (strength) of the 2 sex groups distinguished by sleep health network analysis. (A) Women’s network. (B) Men’s network. At the top of the 2 tables, nonrestorative sleep has the highest centrality in the 2 networks. At the bottom, coffee use has the lowest centrality. Color groupings are only given for data visualization purposes. A higher-resolution version of this image is available in Multimedia Appendix 4.

Predictability results (ie, if a given node could be predicted by adjacent nodes) were interesting when identifying relevant relationships. For instance, in the women’s network, the 6 most central nodes had the following centrality values: nonrestorative sleep, 2.50; excessive daytime sleepiness, 1.70; chronic sleep deprivation, 1.53; chronic circadian misalignment, 1.40; leg sensory discomfort, 1.08; and wakefulness satisfaction, 0.87, as shown in Figure 3. These symptoms were also among the most predictable: predictability values were 0.54, 0.40, 0.68, 0.36, 0.33, and 0.39, respectively (mean 0.26, SD 0.15). All the predictability measures (variance) are given in Multimedia Appendix 2. However, in the women’s network, for nearly identical centrality, chronic sleep deprivation had greater predictability (0.68) than chronic circadian misalignment (0.36), roughly two-thirds to one-third. This result illustrates the need to consider the first of these nodes as being highly predictable but also central to modifying other nodes.

#### Network Centrality

In terms of the local structure, 2 symptoms were found among the 4 most central ones, regarding all the centrality measures of all the networks (for all age and sex groups, see Figure S1 in Multimedia Appendix 1): nonrestorative sleep (belonging to the “sleep disturbances” type) and excessive daytime sleepiness (belonging to the “symptoms of sleep disorders” type). The value of the strength for each measure is given in Multimedia Appendix 2.

This means that these variables exhibited a high degree of connection in the entire sleep network (strength), a high degree of connection with proximal variables of the sleep network after considering negative correlation values (expected influence), the shortest path between 2 other variables (betweenness), and the shortest mean distance from other variables (closeness).

The differences in centrality for the 2 sexes are given in Figure 3. The 3 most important centrality measures for the women’s network were, in descending order, nonrestorative sleep, excessive daytime sleepiness, and chronic sleep deprivation; for the men’s network, these were nonrestorative sleep, chronic circadian misalignment, and excessive daytime sleepiness.

**Figure 4:**
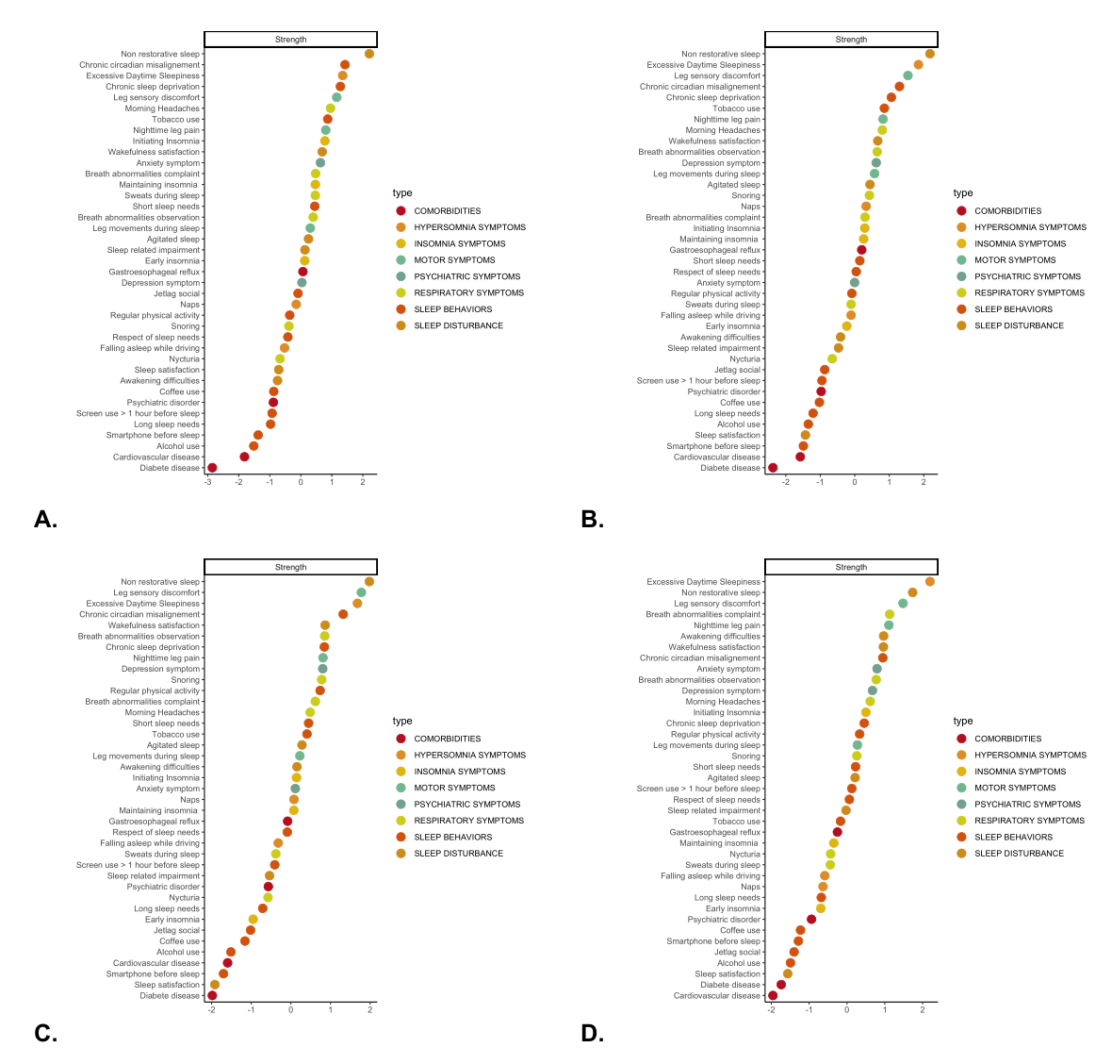
Centrality measure (strength) of the 4 age groups distinguished by sleep health network analysis. (A) 18-30 years, (B) 31-45 years, (C) 46-55 years, and (D) >55 years. At the top of the 4 tables, nonrestorative sleep has the highest centrality for all the groups. At the bottom, sleep satisfaction has the lowest centrality for the 46-55–year group. Color groupings are only given for data visualization purposes. A higher-resolution version of this image is available in Multimedia Appendix 4.

Figure 4 shows the strength (centrality measure) of the 4 age groups in the sleep health network analysis. The 3 most important centrality measures for the 18-30–year network were, in descending order, nonrestorative sleep, chronic circadian misalignment, and excessive daytime sleepiness; for the 31-45–year network, these were nonrestorative sleep, excessive daytime sleepiness, and leg sensory discomfort; for the 46-55–year network, these were nonrestorative sleep, leg sensory discomfort, and excessive daytime sleepiness; and for the >55-year network, these were excessive daytime sleepiness, nonrestorative sleep, and leg sensory discomfort.

Nonrestorative sleep and excessive daytime sleepiness were among the 3 most central symptoms in all 4 groups (strengths were 1.73-2.21 and 1.35-2.19, respectively). However, in the 18-30–year group, the 2 other most central symptoms were chronic circadian misalignment (strength=1.42) and chronic sleep deprivation (strength=1.27), particularly in the women’s network, belonging to the “sleep behaviors” type. Between the ages of 31 and 55 years, the 2 other most central symptoms were chronic circadian misalignment (strength=1.30 for the 31-45–year group and strength=1.32 for the 46-55–year group) and leg sensory discomfort (strength=1.54 for the 31-45–year group and strength=1.78 for the 46-55–year group, belonging to the “motor symptoms” type. In the older group of participants (>55 years old), leg sensory discomfort (strength=1.48) remained 1 of the most central symptoms, while breath abnormality complaint became among the 4 most central symptoms (strength=1.13, belonging to the “respiratory symptoms” type), becoming more central than sleep behaviors found in the youngest. In Multimedia Appendix 4, we provide and discuss the example of a network with its centrality, including the BMI.

**Figure 5:**
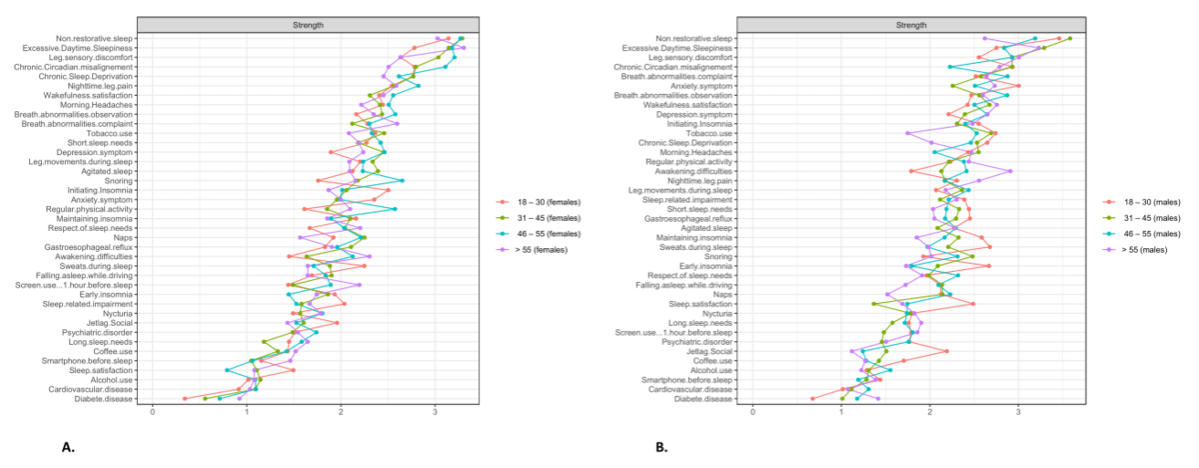
Centrality measure (strength) of the 4 age groups relative to the women’s and men’s networks, distinguished by sleep health network analysis: the 4 age groups for (A) women and (B) men. At the top of the 4 tables, nonrestorative sleep has the highest centrality for the 31-45–year group in men. At the bottom, diabetes disease has the lowest centrality for the 18-31–year group in both women and men. A higher-resolution version of this image is available in Multimedia Appendix 4.

Finally, Figure 5 shows the differences in centrality between the ages according to sexes.

#### Network Comparisons

Here, we present the 2 results of the NCT, comparing (1) the connections between variables and (2) the results concerning centrality (ie, global strength). All the comparisons between variable connections for the 2 networks (men and women) were significantly different (*M* test: 0.100, *P*<.001). However, there were no differences in terms of centrality (global strength of the women’s network=15.27, global strength of the men’s network=13.34; *S* test: 1.92, *P*=.68).

In the same way, as shown in Table 2, all the comparisons between variable connections for the 4 age networks were significantly different. However, as shown in Table 3, no significant difference in terms of centrality measures was found. Multimedia Appendix 3 presents the local symptom-by-symptom correlations (*P* value based on permutations) between connections and strength on sexes and all age groups.

**Table 2:** Difference in connections between age groups (*M* test and *P* value) of the French-speaking adult population concerned about their sleep (N=35,808).

Age group (years)			18-30	31-45	46-55	>55
18-30			—^a^	—	—	—
**31-45**						
	*M* test	0.057		—	—	—
	*P* value	.01		—	—	—
**46-55**						
	*M* test	0.087		0.061	—	—
	*P* value	<.001		.04	—	—
**>55**						
	*M* test	0.159		0.122	0.083	—
	*P* value	<.001		<.001	<.001	—

^a^Not applicable.

**Table 3:** Difference in centrality (global strength) between age groups of the French-speaking adult population concerned about their sleep (N= 35,808).

Age group (years)			18-30	31-45		46-55		>55
18-30			—^a^	—		—		—
**31-45**								
	Strength^b^	12.78 vs 12.23		—	—		—	
	*S* test	0.569		—	—		—	
	*P* value	.37		—	—		—	
**46-55**								
	Strength	12.76 vs 11.54		12.19 vs 11.54	—		—	
	*S* test	1.213		0.643	—		—	
	*P* value	.69		.92	—		—	
**>55**								
	Strength	12.76 vs 12.28		12.19 vs 12.28	12.28 vs 11.54		—	
	*S* test	0.477		0.092	0.735		—	
	*P* value	.89		.99	.41		—	

^a^Not applicable.

^b^Strength in the networks in columns versus strength in the networks in rows.

#### Network Robustness

The robustness of strength is shown in Figure S2 in Multimedia Appendix 1. Robustness was judged acceptable, with a CS coefficient for strength at 0.91 for the women’s network; at 0.75 for the men’s network, the 18-30– and 31-45–year networks; at 0.672 for the 46-55–year network; and at 0.75 for the >55-year network.

## Discussion

### Principal Findings

Our study built on initial work describing key variables in people’ experience of their sleep complaints, in a population that was sufficiently concerned about their sleep to use the Réseau Morphée online sleep questionnaire. We showed that in network analysis on clusters defined by sex or age, the symptoms nonrestorative sleep and excessive daytime sleepiness and an element of sleep behavior (circadian irregularity) remained central to all groups. There were no statistical differences in these elements between the groups, implying that the underlying processes that drive the network and the underlying symptom structure are the same in men and women and the same across different age groups. However, differences in internodal connections between the clusters mean that sleep complaints are not connected in the same way in each of them. This difference should be interpreted as a difference in terms of the structure and the hierarchy in interactions between symptoms. For instance, in the women’s network, the symptoms breath abnormality observation, breath abnormality complaint, and snoring are much less related than they are in the men’s network (see Figure 1).

### Comparison With Prior Work

The elements identified as central to the network for both men and women across all ages are best understood as the impact of underlying processes on the experience of sleep. As discussed in the *Introduction* section, the basic neuroanatomy underpinning sleep is identical in men and women, and although sex-related differences exist and functions may diminish with age, the structure remains. This leads to an important conclusion about improving sleep at a population level. For interventions to be effective in men and women and across all age groups, it is necessary to target central elements with high predictability: high predictability means that interventions aimed at these variables have a high chance of influencing the variables around them [70]. Fortunately, all 3 central variables showed relatively high predictability: interventions aimed at the key sleep-related behaviors have thus a high chance of working in men and women of all ages and of positively influencing other elements of the network.

Excessive daytime sleepiness is due to hypofunction of the wake system. This may be due to neuroanatomical modifications (eg, neuronal loss in the elderly or in patients with narcolepsy) or due to the effect of sleep fragmentation (eg, in obstructive sleep apnea) with incomplete recovery of the homeostatic system. Excessive daytime sleepiness has been shown to be central in both the *International Classification of Sleep Disorders, Third Edition* (ICSD-3) symptom network [40] and in the sleep-wake disorders symptom network of the *Diagnostic and Statistical Manual of Mental Disorders, Fifth Edition* (DSM-5) [41]. Although sensitivity to sleep need may decrease with age and excessive daytime sleepiness is more common in women [75], the centrality of excessive daytime sleepiness as a consequence of impaired sleep or the wake system or both remains across all clusters.

Nonrestorative sleep, defined as a “subjective feeling of being unrefreshed upon awakening,” is considered a result of poor-quality or unrestful sleep. This symptom is poorly understood and has been little researched [6]. Ohayon [38] reported that nonrestorative sleep was present in 10.8% of a European sample and 11.4% of French-speaking respondents. Nonrestorative sleep is found to be more common in women and younger subjects, increased in patients with psychiatric disorders, and much more frequent in people dissatisfied by their sleep and those with difficulty getting started in the morning. It is also associated with excessive daytime sleepiness, which would confirm our findings related to the centrality of nonrestorative sleep and excessive daytime sleepiness. However, the relationship between these 2 elements differs with sex: there is a direct and positive link between the 2 elements in women, whereas in men, the 2 nodes are more distant from each other and less strongly linked. The link also varies with age, with excessive daytime sleepiness and nonrestorative sleep being closer together and more strongly linked in middle age compared to young age (18-30 years) and older persons (>55 years). It is tempting to see nonrestorative sleep as an element related to the homeostatic system, with a residual sleep need at wakening due to either sleep disorders or circadian misalignment. The maintenance of its centrality across the different clusters is interesting. It clearly underpins the experience of sleep, and further research on its nature and correlates is long overdue.

Finally, a behavior-related variable, circadian irregularity, retained its centrality across all clusters. Circadian irregularity relates to irregular sleep timing, with the risk that the actual timing of sleep is out of phase with the circadian rhythm. This is termed “circadian misalignment” and has been recently identified as 1 of the early indicators of cardiometabolic and neurological disorders. It is interesting to note that the central common features reunite physiological elements that underpin sleep: the homeostatic system, behavior affecting the circadian system, and the consequences of poor sleep. However, the centrality of circadian irregularity is particularly important as it is a clear target for behavioral interventions involving sleep: programs encouraging regular sleep are likely to positively influence the experience of sleep across the population.

After the homogeneity of the 3 central variables, the clusters differed in the subsequent variables: sleep-related respiratory and motor symptoms are more frequent in the older age groups. Sleep disorders, notably sleep apnea, are known to increase with age [30]. In the younger age group, sleep-related behaviors are more central, with chronic sleep deprivation being particularly important in young women.

Although no overall differences were detected for centrality, there were clear differences in the connectedness of the networks, which implies that the links between the different nodes are different: that is, although the structure of the network may be the same, the strength of the links between the different nodes is influenced by age and sex. This is seen in the relationship between excessive daytime sleepiness and nonrestorative sleep, where the relationship is closer and more direct in middle age compared to young age or those >55 years old (eg, the *P* value was highly significant between the 18-30– and >55-year groups; *P*<.001).

Global strength increased in women and also in the 45-55–year group. The increase in global strength relates to the fact that all the variables are more closely related. This indirectly implies increased connectedness in the network: sleep symptoms, sleep behaviors, and sleep disorders are all more closely interlinked in women than in men or in older or younger age groups.

### Limitations

Our study has clear limitations. First, we did not consider gender but rather considered sex as assigned at birth, which, despite the lack of consideration for the choice of the person involved, can improve epidemiological comparability. Second, our population comprises French-speaking participants who were concerned about their sleep and is thus not representative of the general population. Despite the large size of our sample, we should therefore be cautious about the generalizability of these results to the general population. Due to anonymity regarding certain sociodemographic data, we cannot provide the location of the participants’ primary residence. The fact that the average ISI, HADS-A, and HADS-D scores exceeded their respective thresholds indicates that on average, participants in our study displayed elevated scores for insomnia, anxiety, and depression. These characteristics may limit the representativeness (eg, whether participants were selected or self-selected based on certain criteria) or overall interpretation of the results (eg, the need to create subgroups). Moreover, an increase in nonrestorative sleep was noted in patients dissatisfied with their sleep in a study by Ohayon [76], and the interpretation of our results should bear this in mind. Third, participants responded to an online questionnaire with a structured symptom and sleep behavior list rather than a validated questionnaire: although this limited comparisons with other studies, it did, however, permit the inclusion of items such as screen use, which are often lacking from older questionnaires [41]. Finally, network analysis is a new and powerful way of analyzing large data sets, but interpretation of results and, notably, centrality should be performed with care. Network analysis highlights the dense interaction between variables and their interdependence at a population level but not at an individual level.

### Future Research Direction

The results of this study provided 3 main perspectives. First, at the clinical level, this research advances our understanding of sleep variations by age and sex, centering on symptoms rather than diagnoses. Second, at the methodological level, it explores an area often underrepresented in the literature but essential for clinical practitioners, providing a weighted visualization of symptom relationships of sleep health, which is important when considering treatments such as cognitive-behavioral therapies. Finally, the study helps promote public health strategies, emphasizing the importance of the underlying unity of sleep health, while promoting a tailored and precise strategy for specific subgroups.

### Conclusion

To conclude, this symptom network analysis on sleep health across various age and sex demographics demonstrates the stability of sleep health across sex and age groups, while highlighting the centrality of nonrestorative sleep and excessive daytime sleepiness. More precisely, younger individuals, especially women, show a higher centrality of behavioral-related sleep issues, whereas the older group has more sleep-related respiratory and motor symptoms. These findings emphasize the need for both general and tailored sleep promotion and screening strategies based on age and sex to effectively address and enhance sleep health in the broader population. Furthermore, it paves the way for health care professionals to adopt a more individualized approach in assessing and treating sleep disorders, thereby enriching the overall quality of patient care.

## Appendix

Multimedia Appendix 1 Supplementary material 1.

Multimedia Appendix 2 Supplementary material 2.

Multimedia Appendix 3 Supplementary material 3.

Multimedia Appendix 4 Higher-resolution images of Figures 1-5.

Multimedia Appendix 5 Supplementary material 4.

## Abbreviations

CS: correlation stability
ESS: Epworth Sleepiness Scale
HADS-A: Hospital Anxiety and Depression Scale for anxiety
HADS-D: Hospital Anxiety and Depression Scale for depression
ISI: Insomnia Severity Index
MGM: mixed graphical model
NCT: network comparison test
SCN: suprachiasmatic nucleus
VIP: vasoactive intestinal peptide

## Data Availability

Data can be provided upon request from the corresponding author.
