# Supplementary material for: Sleep Health Analysis Through Sleep Symptoms in 35,808 Individuals Across Age and Sex Differences: Comparative Symptom Network Study
Source: JMIR Public Health Surveill. 2024 Jun 11;10:e51585. doi: 10.2196/51585 (PMC11200043; doi:10.2196/51585)
Supplement: Multimedia Appendix 1 [file publichealth_v10i1e51585_app1.docx]

**Sleep Health Analysis Through Sleep Symptoms in 35,808 Individuals Across Age and Sex Differences: A Comparative Symptom Network Study**

## **Multimedia Appendix 1 (Supplementary Material 1)**

## **Introduction to network analysis**

A network analysis representing all items and connections between them was constructed graphically (network estimation), quantified with classical metrics (network inference with global measures, i.e. clustering coefficient and average shortest path length and local measures, i.e. centrality measures) and tested for network robustness (in terms of accuracy of edge-weights and stability of centrality measures) [60],

## **Network estimation and accuracy**

A main advantage of the network approach is that it visualizes the multivariate dependencies of the data that would otherwise remain hidden. The most informative visualization is one in which edges can be understood as partial correlation coefficients, meaning that a connection between nodes is the connection after controlling for all other edges in the network [48].

An Ising model can be understood as estimating partial correlations among a set of binary items. It comprises two important processes: the estimation of pairwise relationships between the variables (i.e., the edges) and a network regularization (i.e., to consider the entire network), limiting the number of non-null edges according to sample size. The estimation of pairwise relationships between the variables can be seen as a conditional dependence relation. An association between two variables means that they remain conditionally dependent after controlling for all other associations among the variables in the global network. Conversely, if no edge emerges between two variables, they are conditionally independent after controlling for the associations among all other variables.

In order to minimize the risk of false positives (i.e., a nonzero connection estimated although there is no actual connection), we used a regularization technique called graphical least absolute shrinkage and selection operator [58,59]. The LASSO penalizes the sum of absolute parameter values such that the estimated values shrink to zero. A tuning parameter lambda (λ) provides different estimating network structures for various lambda values. The final model is chosen by using the extended Bayesian information criterion (EBIC) [60]. The result is a sparse and conservative network, in which connections that are estimated to be present (i.e., nonzero) are likely true positives, whereas the connections that are estimated to be absent (i.e., zero) may be false negatives.

Based on this estimation, a correlation matrix was obtained with Pearson correlations (in order to show side by side the association of two variables in the diagnostic criteria of sleep disorders).

The network was graphically represented according to a Fruchterman-Reingold algorithm [61]. In this graphic representation, the weight of the connection between two nodes is proportional to the correlation measures, and the place of the node is positioned according to a force-directed graph measure, so that variables with stronger and/or more connections are placed closer to each other.

We then computed the predictability of a node, depicted as a pie chart in the rings around nodes. Predictability answers the question of how well a given node in the network can be predicted by all remaining nodes (the pie argument has a value between 0 and 1 for each node). Predictability is interesting for knowing how practically relevant edges are (e.g., if a node is connected to many other nodes but these only explain only 1% of its variance, how interesting are the edges connected to A?), how to design an intervention in order to achieve a change in a certain set of nodes, or which different parts of the network are (self-) determined by other factors that are not included in the network. Predictability of nodes within a network are computed based on models derived from Mixed Graphical Models (mgm) [62], using a node-wise regression approach to estimate the network structures. Predictability is calculated on the base of the aggregated output of two different estimation methods: the regularized partial correlation network of the LASSO and EBIC model selection, described above, and the mgm estimation.

## **Network inferences: global measures**

The global measures of a network are related to small-world measures, which are measured using the clustering coefficient (degree to which nodes in a graph tend to cluster together) and the average shortest path length (the average over the shortest path lengths of all node pairs) [69].

The clustering coefficient can be approximated to three times the number of triangles divided by the number of connected triples of vertices. The shortest path length between two nodes equals the minimum number of edges that must be passed over to get from one to the other. The average shortest path length is the average over the shortest path lengths of all node pairs. A small world will exhibit the following global network properties: a higher clustering coefficient and average shorter path lengths than in a random network (i.e., a network whose distribution can be described by a random process). Clustering-Sleep and Average Path Length (APL-Sleep) were calculated on the sleep network. Clustering-Random and APL-Random were calculated on a network with the same number of nodes connected by the same number of edges as this sleep network. The sleep network small-worldness index (SWI) was then calculated according to the following formula: SWI = (Clustering-Sleep / Clustering-Random) / (APL-Sleep / APL-Random). The SWI should exceed the conservative small-worldness criterion threshold of 3 with the “HG” measure (according to the HG-scale – [49]), a SWI > 3 indicates a small-world network). Small-world measures for networks may be used to evaluate the degree of association between variables. This helps clinicians to rapidly look for the other variables of a community (e.g., a syndrome) (known in network theory as “high signal-propagation speed”), and the global consistency and manipulability of the diagnostic manual for clinicians (known as “computational power”).

## **Network inferences: local measures**

The local measures of a network are related to centrality measures [67]. Centrality measures are important for identifying bridge variables, which play a crucial role in connecting two or more sleep disorders [68]. The centrality parameters of a network are particularly important to better understand the relationship between variables in a patient. Nodes with high centrality index measures represent variables that are highly connected to other variables. The correlations between variables may be considered as stronger when the nodes have higher centrality. In the network graph (using Fruchterman-Reingold algorithm), central nodes often end up in the center of the graph and nodes with low centrality in the periphery [61]. These centrality statistics can be quantified by four measures: Strength, Closeness, Betweenness and Expected Influence [69].

The strength of a node computes the weighted number of connections for a given node, and therefore the degree to which it is connected with all the other nodes of the network [70]. A variable has high strength centrality if this variable is highly connected to all the other variables. In a rail network metaphor, a city has high strength if it is connected to an extremely large number of other cities, e.g., Paris in France.

The closeness of a node is computed according to the shortest path length measure and is inversely proportional to the shortest mean distance from all the other nodes [67]. A variable has high closeness centrality if the variable can be connected shortly to other variables [50,51]. Using the rail network metaphor, a city has high closeness if it is central compared to many other cities, i.e., it is “close” to many others, like the city of Bourges which is very central in France.

The betweenness of a node computes the degree to which a given node acts as a “bridge” connecting different parts of the network, thus reflecting the degree to which it controls the flow of information across the network. A variable has high betweenness centrality if the variable can influence the connection between non-adjacent (i.e. not directly connected) variables, thus acting as gatekeeper [52]. In the railroad network metaphor, a city has high betweenness if it is necessary to transit through that city to reach other cities, like the city of Lyon which must be passed through on the route between Paris and Marseille.

The Expected Influence of a node is computed to improve the measurement of centrality of the nodes in a network [53]. It is computed as the sum of all edges which extend from a given node, accounting for both positive and negative correlation values with regard to the entire network. A variable with high Expected Influence has an influence on the variable network based on its positive correlations, negative correlations being corrected by this centrality measure. In the railroad network metaphor, a city has a high expected influence if it is connected to an extremely large number of connected neighboring cities, considering possible negative correlation values between cities.

## **Network robustness**

When estimating psychopathological networks, a major current challenge is that the stability of centrality measures of the psychopathological networks are unclear [73,74].

In order to verify that the number of subjects was adequate to perform such a network analysis, we analyze the robustness of the network by a bootstrap analysis (N=2,000 iterations), with the use of a case-dropping subset assess the stability of centrality indices (providing a centrality stability correlation coefficient – CS-coefficient). We used a case-dropping subset bootstrap to assess the stability of centrality indices, and more precisely one of them, the strength. We aim to show how well the order of strength is retained after observing only a subset of the data. This stability can be quantified using the centrality stability correlation coefficient (CS-coefficient), which represents the maximum proportion of participants that can be dropped while maintaining 95% probability that the correlation between centrality metrics from the full data set and the subset data are at least 0.70. Based on a simulation study [54,55], a minimum CS-coefficient of 0.25 is recommended. If correlation values decline substantially as participants are removed, then this centrality index would be considered less stable [55].

## **Computational Analysis**

Processing and graphical visualizations used the R (4.1.0) package bootnet (version 1.2.3) [55,69], which leads more strongly connected sets of nodes to cluster closer together than the R-packages MGM (version 0.3.1) [56], Pearson correlations for the binary data in our dataset and with the qgraph package for visualization (version 1.6.3) Expected influence values were calculated with the networktools R package. The robustness of the results is tested on edge-weights and centrality measures [55]. Finally, the small-worldness index was calculated with the NetworkToolBox [57], with generation of 2,000 random networks and the “HG” measure from Humphries and Gurney [49]. According to previous work and from the point of view of clinical relevance, only positive edges were considered in the small-worldness index. The codes and data are freely accessible on demand.

## **Results of centrality**

Centrality measures of the four age groups are given in the Figure S1.

**A. B.**

**Figure S1**. Centrality measure in terms of Expected Influence, Betweenness and Closeness of the network analysis (ordered by Strength), for sexes (A.) and age groups (B.)

## **Results of robustness**

Robustness is given in **Figure S2**.

**Figure S2**. Case-dropping bootstrap procedure for stability of centrality indices in terms of strength, betweenness and closeness.

## **References**

1. Epskamp S, van Borkulo CD, van der Veen DC, Servaas MN, Isvoranu A-M, Riese H, Cramer AOJ. Personalized Network Modeling in Psychopathology: The Importance of Contemporaneous and Temporal Connections. Clin Psychol Sci 2018 May;6(3):416–427. PMID:29805918 50

2.

3. Friedman J, Hastie T, Tibshirani R. Sparse inverse covariance estimation with the graphical LASSO. Biostatistics (Oxford, England) 2008 Aug 1;9:432–41. doi: 10.1093/biostatistics/kxm045 48

4. Tibshirani R. Regression Shrinkage and Selection via the Lasso. Journal of the Royal Statistical Society Series B (Methodological) [Royal Statistical Society, Wiley]; 1996;58(1):267–288. 49

5. Fruchterman TMJ, Reingold EM. Graph drawing by force-directed placement. Software: Practice and Experience 1991 Nov;21(11):1129–1164. doi: 10.1002/spe.4380211102 51

6. Haslbeck JMB, Fried EI. How predictable are symptoms in psychopathological networks? A reanalysis of 18 published datasets. Psychol Med 2017 Dec;47(16):2767–2776. PMID:28625186 52

7. Epskamp S, Cramer AOJ, Waldorp LJ, Schmittmann VD, Borsboom D. qgraph: Network Visualizations of Relationships in Psychometric Data. Journal of Statistical Software 2012;48(4). doi: 10.18637/jss.v048.i04 59

8.

9. Boccaletti S, Latora V, Moreno Y, Chavez M, Hwang D. Complex networks: Structure and dynamics. Physics Reports 2006 Feb;424(4–5):175–308. doi: 10.1016/j.physrep.2005.10.009 57

10. Cramer AOJ, Waldorp LJ, Maas HLJ van der, Borsboom D. Comorbidity: A network perspective. Behavioral and Brain Sciences 2010 Jun;33(2–3):137–150. doi: 10.1017/S0140525X09991567 58

11. Barrat A, Barthélemy M, Vespignani A. Weighted Evolving Networks: Coupling Topology and Weight Dynamics. Physical Review Letters 2004 Jun;92(22):228701. doi: 10.1103/PhysRevLett.92.228701 60

12.

13.

14.

15.

16. Fried EI, Epskamp S, Nesse RM, Tuerlinckx F, Borsboom D. What are “good” depression symptoms? Comparing the centrality of DSM and non-DSM symptoms of depression in a network analysis. Journal of Affective Disorders 2016 Jan;189:314–320. doi: 10.1016/j.jad.2015.09.005 63

17. Borsboom D, Robinaugh DJ, Rhemtulla M, Cramer AOJ. Robustness and replicability of psychopathology networks. World Psychiatry 2018 Jun;17(2):143–144. PMID:29856550 64

18.

19.

20.

21.
