# Supplementary material for: Sleep Health Analysis Through Sleep Symptoms in 35,808 Individuals Across Age and Sex Differences: Comparative Symptom Network Study
Source: JMIR Public Health Surveill. 2024 Jun 11;10:e51585. doi: 10.2196/51585 (PMC11200043; doi:10.2196/51585)
Supplement: Multimedia Appendix 2 [file publichealth_v10i1e51585_app2.docx]

**Sleep Health Analysis Through Sleep Symptoms in 35,808 Individuals Across Age and Sex Differences: A Comparative Symptom Network Study**

## **Multimedia Appendix 2 (Supplementary Material 2)**


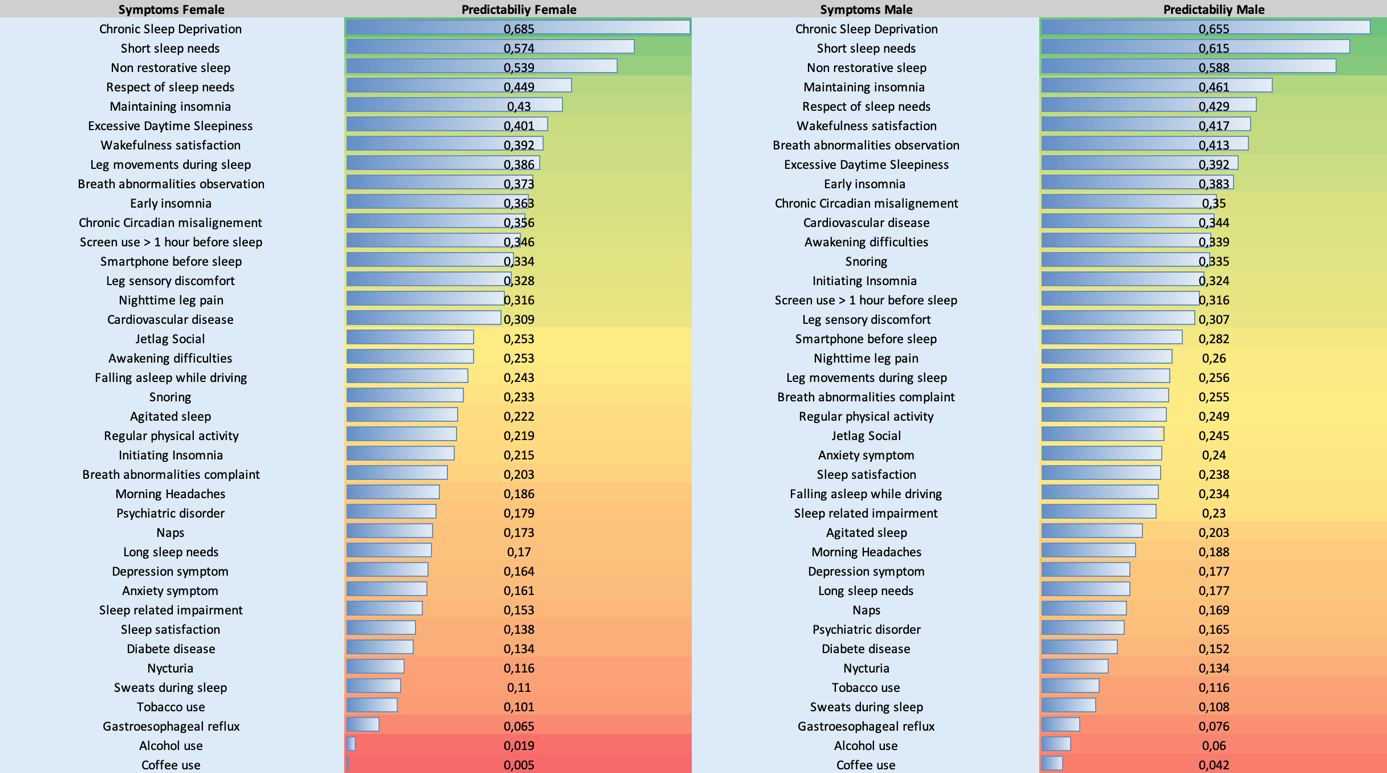


Predictability Sexes


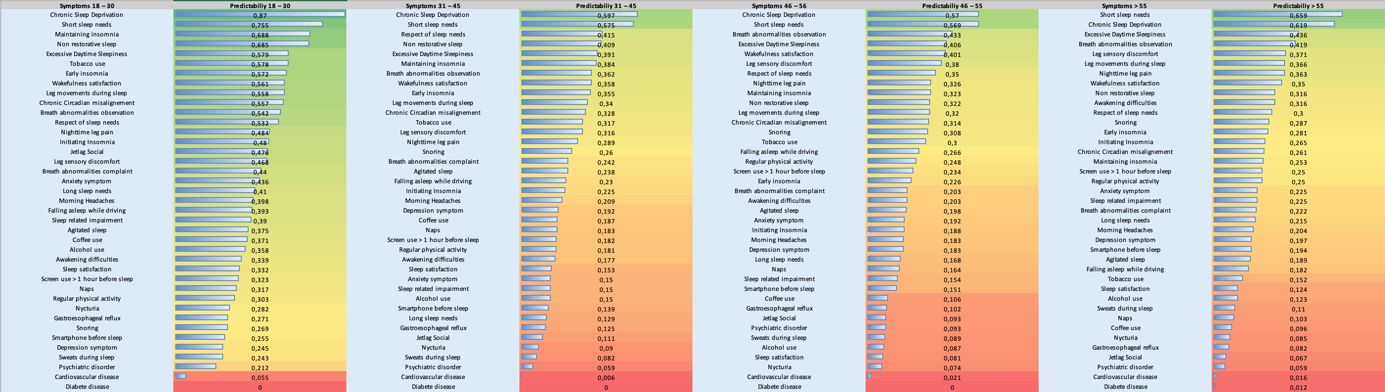


Predictability Age Groups


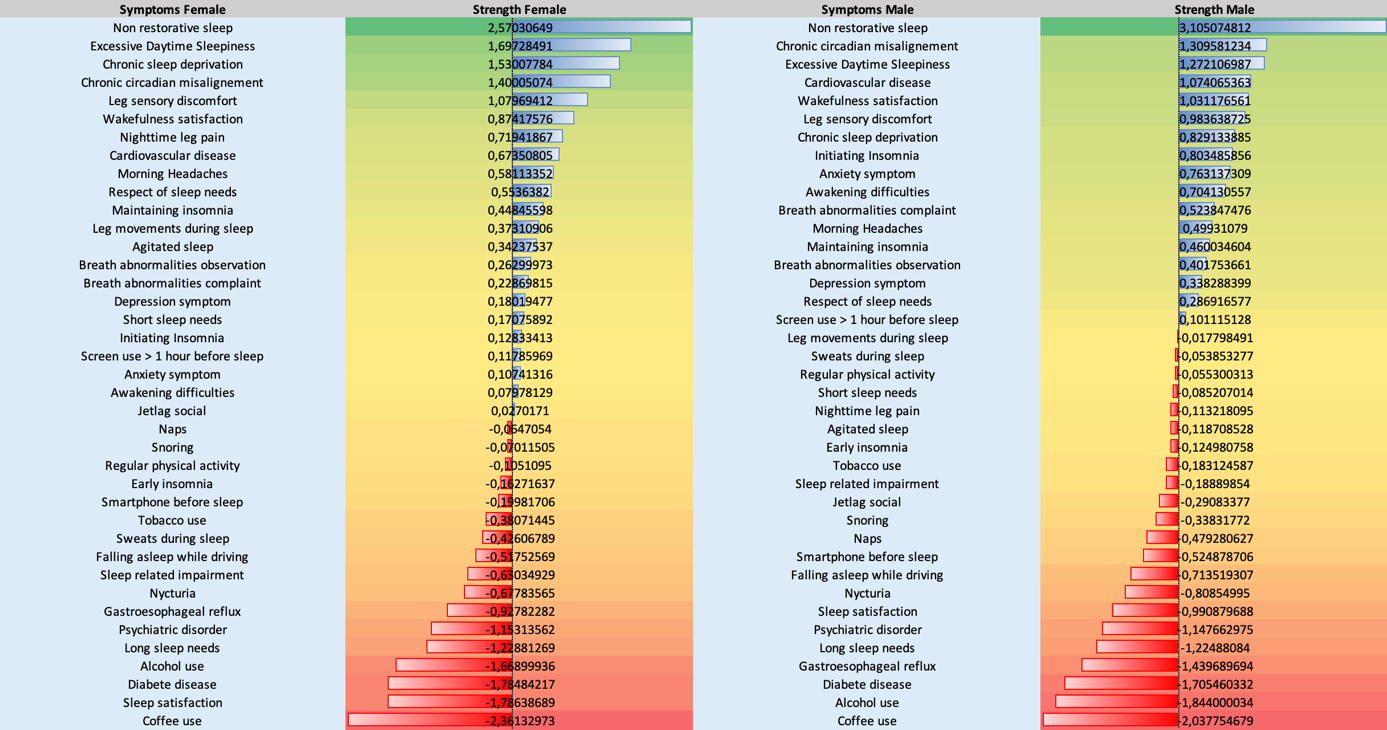


Strength Sex


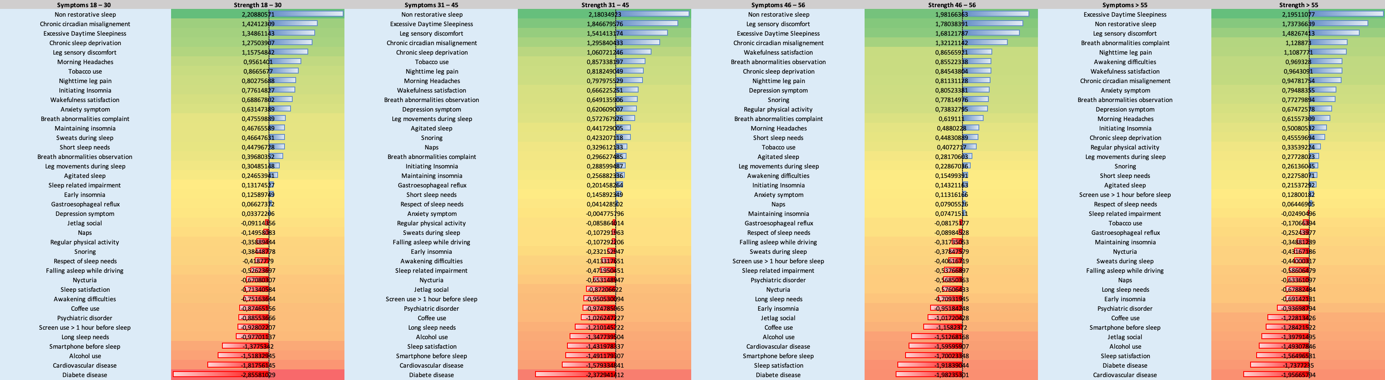


Strength Age Groups
