# Supplementary material for: Sleep Health Analysis Through Sleep Symptoms in 35,808 Individuals Across Age and Sex Differences: Comparative Symptom Network Study
Source: JMIR Public Health Surveill. 2024 Jun 11;10:e51585. doi: 10.2196/51585 (PMC11200043; doi:10.2196/51585)
Supplement: Multimedia Appendix 3 [file publichealth_v10i1e51585_app3.docx]

**Sleep Health Analysis Through Sleep Symptoms in 35,808 Individuals Across Age and Sex Differences: A Comparative Symptom Network Study**

## **Multimedia Appendix 3 (Supplementary Material 3)**


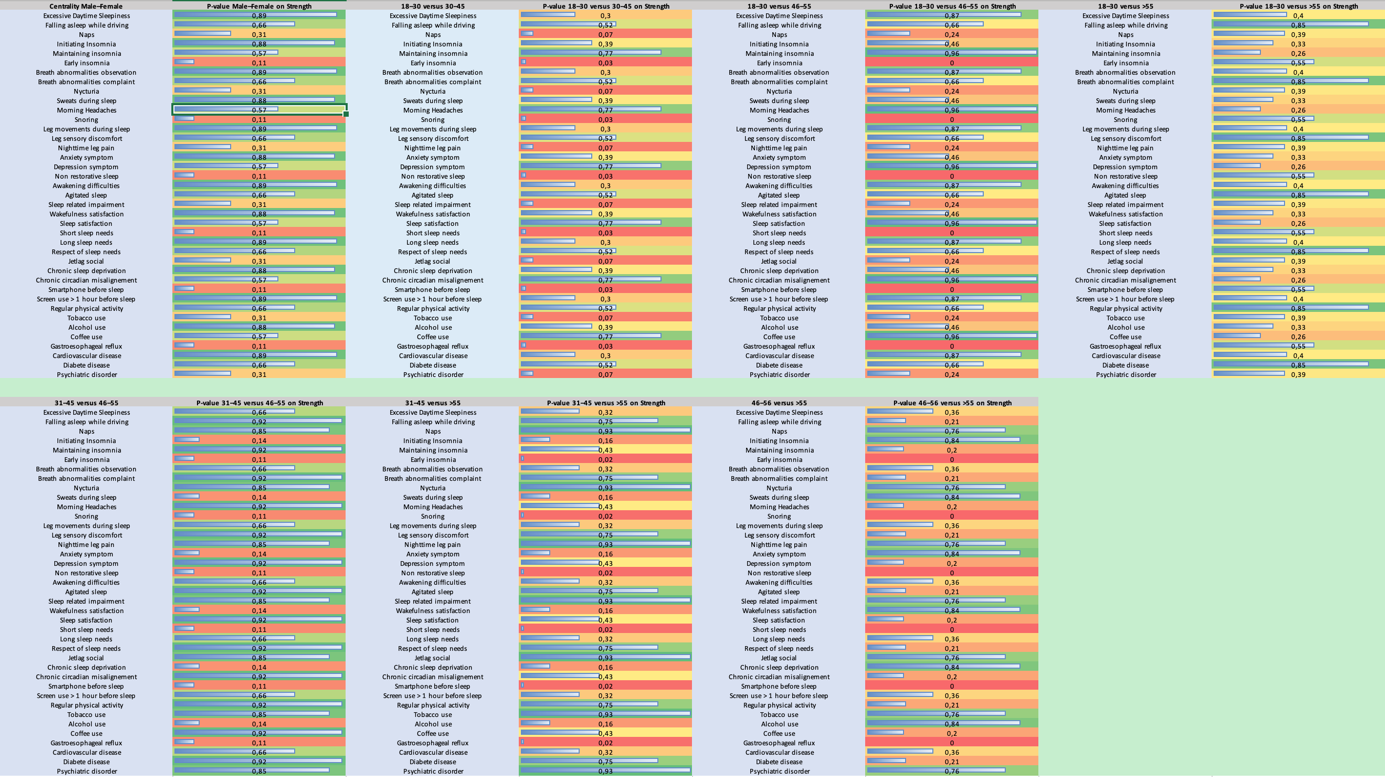


P-Value of Centrality
