# Supplementary material for: Sleep Health Analysis Through Sleep Symptoms in 35,808 Individuals Across Age and Sex Differences: Comparative Symptom Network Study
Source: JMIR Public Health Surveill. 2024 Jun 11;10:e51585. doi: 10.2196/51585 (PMC11200043; doi:10.2196/51585)
Supplement: Multimedia Appendix 4 [file publichealth_v10i1e51585_app4.docx]

**Multimedia Appendix 5: High resolution images of Figures 1-5.**

**Figure 1.** Sleep networks related to sex groups based on a French-speaking adult population (N=35,808) of this cross-sectional comparative network analysis study on sleep health conducted between 2017 and 2020. A. Women’s network. B. Men network. Blue edges (connections) represent the positive associations between variables and orange edges the negative associations. The thickness of the line represents the level of correlation between two variables. Predictability of a node is depicted as a pie chart in the rings around nodes: the area in the outer ring of nodes represents the percentage of variance of the node that is explained by all neighbouring nodes. The colour groupings are only given for data visualization purposes.

**Figure 2.** Sleep networks related to the four age groups, based on a French-speaking adult population (N=35,808) of this cross-sectional comparative network analysis study on sleep health conducted between 2017 and 2020. A. 18 to 30 years old. B. 31 to 45 years old. C. 46 to 55 years old. D. More than 55 years old. Blue edges (connections) represent the positive associations between variables and orange edges the negative associations. The thickness of the edges represents the level of correlation between two variables. Predictability of a node is depicted as a pie chart in the rings around nodes: the area in the outer ring of nodes represents the percentage of variance of the node that is explained by all neighbouring nodes. The colour groupings are only given for data visualization purposes.

**Figure 3.** Centrality measure (strength) of the two sex groups distinguished by sleep network analysis. A. Female network. B. Male network. At the top of the two tables, “Non-restorative sleep” has the highest centrality in the two networks. At the bottom, “Coffee use” has the lowest centrality. The colour groupings are only given for data visualization purposes.

**Figure 4.** Centrality measure (strength) of the four age groups distinguished by sleep network analyses. A. 18 to 30 years old. B. 31 to 45 years old. C. 46 to 55 years old. D. More than 55 years old. At the top of the four tables, variable “Non-restorative sleep” has the highest centrality for all the groups. At the bottom, the variable “Sleep satisfaction” has the lowest centrality for the 46 to 55 age group. The colour groupings are only given for data visualization purposes.

**Figure 5.** Centrality measure (strength) of the four age groups relative to the female and male groups, distinguished by sleep network analyses. A. The four age groups for women. B. The four age groups for men. At the top of the four tables, variable “Non-restorative sleep” has the highest centrality for the 31 – 45 age group in men. At the bottom, variable “Diabetes disease” has the lowest centrality for the 18 to 31 age group in women and men.
