# Supplementary material for: Sleep Health Analysis Through Sleep Symptoms in 35,808 Individuals Across Age and Sex Differences: Comparative Symptom Network Study
Source: JMIR Public Health Surveill. 2024 Jun 11;10:e51585. doi: 10.2196/51585 (PMC11200043; doi:10.2196/51585)
Supplement: Multimedia Appendix 5 [file publichealth_v10i1e51585_app5.docx]

**Sleep Health Analysis Through Sleep Symptoms in 35,808 Individuals Across Age and Sex Differences: A Comparative Symptom Network Study**

## **Multimedia Appendix 4 (Supplementary Material 4)**

We conducted the network analysis by adding the Body Mass Index (BMI), in order to specifically study its impact on sleep health.

At the local level, an interesting result emerges since the BMI is directly strongly correlated, with good predictability, with at least the symptoms of “falling asleep while driving”, “naps” and “leg movements during sleep”. This result should be the subject of additional investigations in future studies, by being compared to the literature.

At the level of the global network structure (see above on the Method section of this Supplementary Material for the differences with the local structure), we remain very cautious about the interpretation of mutual relationships. Indeed, in network analyses, each correlation should be interpreted according to all of the other relationships in the network. It is therefore the measure of centrality that matters. For BMI, the centrality is very low (see the centrality plot below), showing that this variable has very little influence on sleep health.

**Figure S3.** Sleep networks related to sex groups based on a French-speaking adult population (N=35,808) of this cross-sectional comparative network analysis study on sleep health conducted between 2017 and 2020, with the Body Mass Index (BMI) on a specific group.

Figure S4. Centrality measure (strength) of the four age groups distinguished by sleep network analyses, with the Body Mass Index (BMI).

## **Questionnaire**

This questionnaire is given here for informational purposes only. It has not been validated in the English language.

1. Excessive Daytime Sleepiness: How often do you find yourself sleepy during the day?

2. Falling Asleep while Driving: Have you ever felt drowsy or fallen asleep while driving?

3. Naps: Are you bothered by the need to fight the urge to sleep during the day?

4. Initiating Insomnia: Do you find it difficult to initiate sleep at night?

5. Maintaining Insomnia: Once asleep, do you have trouble maintaining sleep throughout the night?

6. Early Insomnia: Do you often wake up earlier than intended and cannot fall back asleep?

7. Breath Abnormalities Observation: Has anyone around you noticed any breathing pauses during the sleep?

8. Breath Abnormalities Complaint: Do you experience or have been told about difficulties in your breathing during sleep?

9. Nycturia: How often do you wake up at night to urinate?

10. Sweats during Sleep: Do you often sweat excessively while sleeping?

11. Morning Headaches: Do you frequently experience headaches upon waking up?

12. Snoring: How often do you snore while sleeping?

13. Leg Movements during Sleep: Are you aware of any leg movements while you sleep?

14. Leg Sensory Discomfort: Do you experience discomfort or restless feelings in your legs at night?

15. Nighttime Leg Pain: Do you suffer from leg pain specifically during nighttime?

16. Anxiety Symptom: We used the Hospital Anxiety and Depression scale 'A' with a threshold of > 10.

17. Depression Symptom: We used the Hospital Anxiety and Depression scale 'D' with a threshold of > 10.

18. Non-Restorative Sleep: How do you usually feel when you wake up? (in shape and ready versus still tired and sleepy)

19. Awakening Difficulties: Do you find it challenging to wake up in the morning?

20. Agitated Sleep: Is your sleep frequently disturbed or agitated?

21. Sleep-Related Impairment: Has lack of sleep impacted your daily functioning?

22. Wakefulness Satisfaction: Are you satisfied with the quality of your wakefulness during the day?

23. Sleep Satisfaction: Are you satisfied with the quality and duration of your sleep?

24. Short Sleep Needs: Do you feel refreshed and functional even with shorter durations of sleep?

25. Long Sleep Needs: Do you feel that you require longer durations of sleep than most people to feel refreshed?

26. Respect of Sleep Needs: Do you regularly ensure that you meet your sleep requirements?

27. Jetlag Social: Do you experience jetlag-like symptoms due to social commitments?

28. Chronic Sleep Deprivation: Have you been consistently getting less sleep than required over an extended period?

29. Chronic Circadian Misalignment: Do you have a consistent mismatch between your internal clock and your sleep-wake cycle?

30. Smartphone before Sleep: How often do you use your smartphone right before sleeping?

31. Screen Use > 1 Hour Before Sleep: Do you often engage with screens for more than an hour before bedtime?

32. Regular Physical Activity: How often do you engage in physical activity?

33. Tobacco Use: Do you use tobacco products, and if so, how frequently?

34. Alcohol Use: How often do you consume alcohol, especially before bedtime?

35. Coffee Use: How many cups of coffee do you typically consume in a day?

36. Gastroesophageal Reflux: Do you experience symptoms of acid reflux, especially at night?

37. Cardiovascular Disease: Have you been diagnosed with any cardiovascular diseases?

38. Diabetes: Have you been diagnosed with diabetes?

39. Psychiatric Disorder: Have you been diagnosed with or are you experiencing symptoms of a psychiatric disorder?
